# Uric Acid in First-Episode Psychosis: A Systematic Review

**DOI:** 10.31083/AP44248

**Published:** 2025-06-25

**Authors:** Daniel Rego, Sofia Ramos Ferreira, Nuno Madeira

**Affiliations:** ^1^Faculdade de Medicina, University of Coimbra, 3000-548 Coimbra, Portugal; ^2^Psychiatry Department, Coimbra Hospital and Universitary Centre, 3000-075 Coimbra, Portugal; ^3^Instituto de Psicologia Médica, Faculdade de Medicina, University of Coimbra, 3004-504 Coimbra, Portugal; ^4^Coimbra Institute for Biomedical Imaging and Translational Research, University of Coimbra, 3000-548 Coimbra, Portugal

**Keywords:** uric acid, first-episode psychosis, schizophrenia, biomarkers

## Abstract

**Background:**

Psychotic disorders such as schizophrenia (SCZ) have been frequently linked with oxidative stress, with uric acid (UA) levels being of particular interest, although evidence remains inconclusive. A possible reduction of UA levels in early phases of SCZ, namely first-episode psychosis (FEP), has been hypothesized. In this systematic review, we aimed to analyze and summarize current evidence regarding UA levels in patients with early psychosis.

**Methods:**

We conducted a systematic review of case-control studies comparing serum or plasma UA levels in individuals experiencing FEP with those in healthy controls (HC).

**Results:**

Eight studies met the inclusion criteria, with a total sample of 950 individuals that included 520 FEP patients and 430 HC. A tendency for lower UA levels in FEP was described, albeit without definitive evidence, and decreased UA levels were restricted to certain ethnic populations.

**Conclusions:**

Our findings do not fully support the hypothesis of an oxidative stress response in early psychosis translatable with reduced UA levels in patients with FEP. Further research is warranted to elucidate the nature and magnitude of the relationship between oxidative stress, UA levels, and early psychosis.

## Main Points

1. Metabolic abnormalities such as altered uric acid (UA) levels have been extensively described in patients with schizophrenia (SCZ), including in first-episode psychosis (FEP).

2. In this systematic review we found no consistent reduction of UA levels in patients with early phases of SCZ, namely FEP; previously reported findings could be restricted to specific subgroups.

3. Longitudinal studies prospectively tracking UA levels in cohorts of FEP patients could elucidate UA’s role as a possible biomarker of affective vs non-affective psychosis.

## 1. Introduction

The onset of psychosis can stem from various factors, including genetics, environmental stressors, substance abuse, or underlying psychiatric conditions. First-episode psychosis (FEP) is an intermediate diagnosis used until the latent clinical entity stabilizes and it is known that most patients with FEP will eventually be diagnosed as having schizophrenia (SCZ) and, more rarely, affective disorders such as bipolar disorder (BD) or unipolar depression with psychotic features [1, 2]. The annual incidence of a FEP is approximately 50 in 100,000 people, while the prevalence of SCZ appears to be about 0.3%–0.7% [3, 4]. Psychotic disorders are associated with significant premature morbidity resulting from contributing factors such as cardiovascular disorders and metabolic complications, associated with decreased daily functioning and an increased risk of mortality in comparison to the general population [4]. Metabolic abnormalities have been extensively described in patients with SCZ and were found in several disease stages, even in drug-naïve patients [5]. Lower prevalences of metabolic dysfunction at the onset of psychotic illness suggests that antipsychotic medication might play a role, and a meta-analytic review about the prevalence of metabolic syndrome in SCZ patients reported that the strongest influence on this outcome was illness duration [6, 7]. Nonetheless, multiple additional metabolic and hormonal changes (e.g., blood concentrations of anterior pituitary hormones) have been documented even in drug-naïve people with first-episode SCZ [5, 8]. It has been proposed that oxidative injury occurs at the onset of psychosis and could be a feature of the disease process itself [1].

Uric acid (UA) is the final oxidation product of the adenine- and guanine-based purine catabolic pathway [9], and a selective antioxidant that removes nitrogen peroxide radicals, playing an important role as a free radical scavenger in the human body [10]. Amongst other metabolic parameters, the predictive value of UA in FEP has been hypothesized as useful to differentiate between non-affective and affective psychosis [11]. Purine metabolism is believed to be one of the mitochondrial antioxidant defense strategies by producing UA and regulating diverse physiological processes, namely mood and sleep; strong evidence supports its participation in the pathogenesis of severe mental disorders [11, 12]. UA is believed to have dual roles in the antioxidant defense system (AODS)—a complex, interrelated system which dampens oxidative stress and protects tissue components from free radical-mediated damage, suggesting UA may exert both neuroprotective or neurotoxic effects in the brain tissue [9], and could possibly be considered as a risk factor in many pathological conditions [9, 13, 14]. It is documented that UA prevents the propagation of oxidative stress from the extracellular to the intracellular milieu by preserving the integrity of the plasma membrane at the lipid-aqueous interface boundary [14]. Thus, decreased UA levels may be indicative of a decreased ability of the body to prevent oxidative stress [14]. Conversely, elevated UA levels are considered markers of an ongoing oxidative stress state related to a heightened purinergic turnover and attenuated adenosinergic transmission [10], which might facilitate the emergence of psychopathological states such as mania and acute psychosis.

There have been multiple studies around metabolic biomarkers, such as UA, in SCZ and even in early phases such as FEP. However, the association between UA levels and different phases of SCZ remain controversial, with studies showing decreased, increased or unchanged UA levels in comparison with healthy controls (HC) [9, 10, 15]. In 2020, a meta-analysis of case-control studies examining UA levels in SCZ subjects (n = 2207) in comparison to those in HC (n = 897) reported unconsistent findings; however, based on a subgroup analysis of 2 studies in FEP patients, it was hypothesized that UA levels could be decreased in subjects with FEP, but not in chronic SCZ [10]. In this systematic review, we aim to assess if individuals with FEP have higher UA levels than HC or comparable clinical conditions.

## 2. Methods

### 2.1 Data Sources and Search Strategy

A systematic search with PubMed database was conducted by two authors using the following Medical Subject Headings (MeSH) terms: (“uric acid” or hyperuricemia or urate or UA or hyperuri* or hypouricemia or hypouri*) and (“first-episode psychosis” or psychosis or “psychotic disorder” or schizophrenia or schizophreni* or “schizophrenic disorders” or “schizophrenic disorder”). PubMed was searched based on its broad overview and MeSH indexing, allowing a controlled and comprehensive searching for medical topics. Preferred Reporting Items for Systematic Reviews and Meta-Analyses (PRISMA) guidelines were followed [16] (**Supplementary Matrial-PRISMA 2020 checklist**).

### 2.2 Inclusion and Exclusion Criteria

Original studies investigating the correlation between UA levels in early psychosis were considered eligible for this systematic review if they met all of the following criteria: (i) publication date between January 2000 and January 2024, (ii) written in English or Portuguese languages, (iii) subjects with FEP or psychotic disorder in an early stage—initial 5 years of disease, (iv) pairwise comparison with a mentally and physically healthy control group and (v) information about the biochemical assays used to measure plasma or serum UA levels. Exclusion criteria included: (i) duplicate reports, (ii) undocumented values of UA for patients and/or control subjects, (iii) lack of control group, (iv) patients with previously diagnosed psychotic disorder, (v) patients who had other psychiatric illness, suffering from endocrinological or cardiovascular diseases.

### 2.3 Data Abstraction

After performing the initial literature searches, each study title and abstract were screened for eligibility by two authors. All potentially relevant studies were fully read and analyzed for eligibility. The PRISMA flow diagram provides detailed information. Data extracted from the included studies were entered into an organized database that included: name of first author, publication year, country, FEP assessment, number of participants with FEP and HC, and UA levels. This review was not previously registered, and its protocol was prepared as is available on request.

## 3. Results

**Fig. 1.**
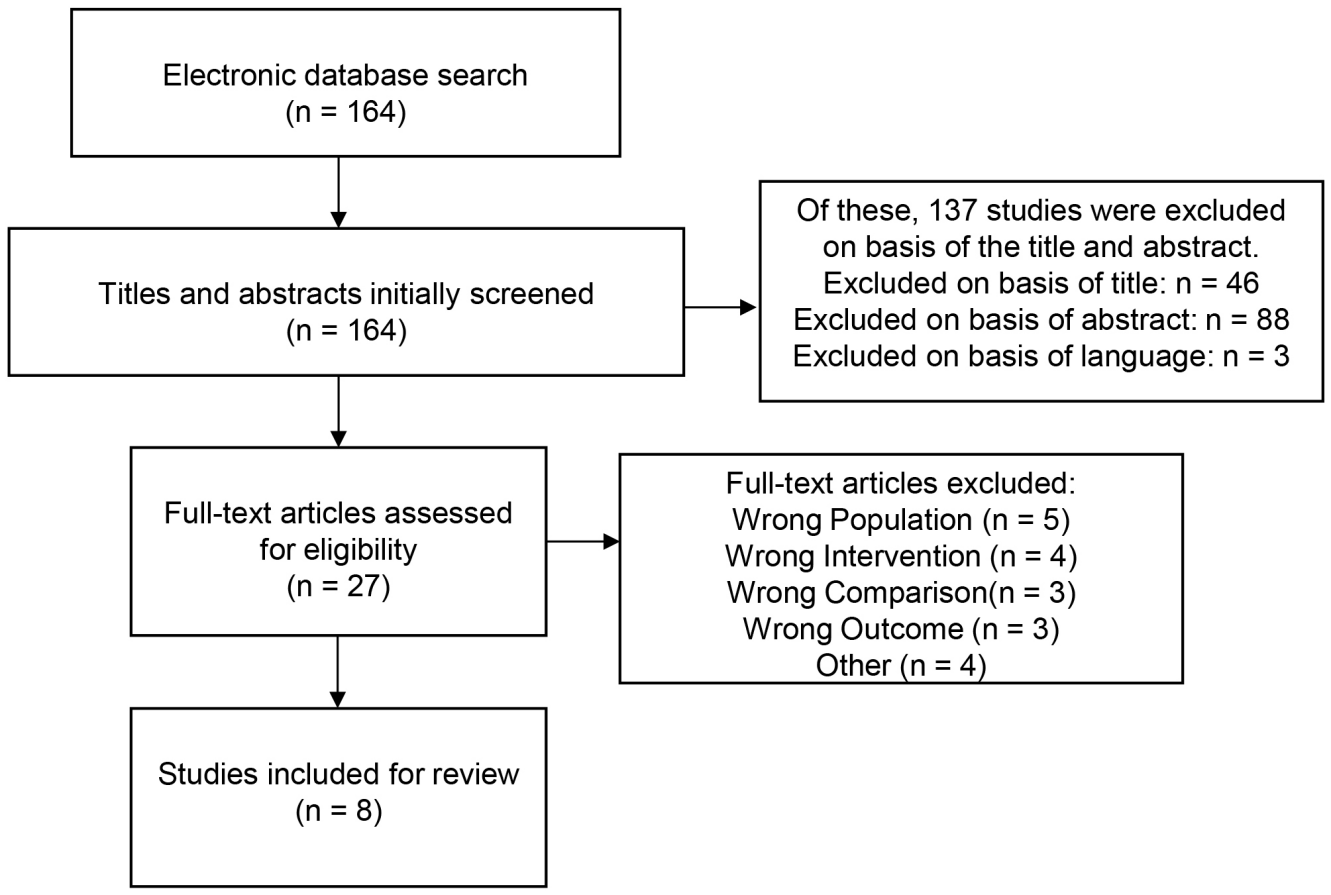
**Preferred Reporting Items for Systematic Reviews and Meta-Analyses (PRISMA) flow diagram of the study selection process**.

A total of 164 records from *PubMed* database were generated by the conducting search. All records have been imported into the reference management tool software Mendeley Reference Manager 2.107.0 (Elsevier, Amsterdam, Netherlands). Of these, 137 studies were excluded based on the title and abstract; the remaining 27 studies were then fully screened. After the final screening, 8 studies met our eligibility criteria and were included in this review [1, 9, 14, 17, 18, 19, 20, 21]. The study selection flowchart is presented in Fig. 1, according to PRISMA guidelines [16].

Therefore, this systematic review included 8 studies, with a total of 950 individuals—520 FEP patients and 430 HC subjects. These studies were conducted in United States of America (USA), South Korea, Türkiye, China and Serbia. UA levels mean ± standard deviation (SD) is given for each patients group and controls group. Gender distribution was slightly skewed towards female patients (53.5%); the median age of the included subjects is 24.9 years. A summary of the results is presented on Table 1 (Ref. [1, 9, 14, 17, 18, 19, 20, 21]).

**Table 1.** **Summary of included studies in the systematic review**.

Reference	Country	Inclusion criteria	Demographic characteristics of FEP patients	Duration of illness	Medication status	Biological sample	UA levels (patients)	UA levels (controls)	Other information
Reddy *et al*. 2003 [14]	USA	DSM-IV for SCZ or SZA	n = 31, M/F = 20/11, Mean age = 28.5 ± 7.8	Not specified	Antipsychotic-naive	Plasma	4.92 ± 1.38 mg/dL	5.76 ± 1.25 mg/dL 5.76 ± 1.25 mg dL (mood disorder patients)	First-episode SCZ or SZA patients had significantly lower UA levels as compared to HC There were no significant differences between first-episode BD or depressive disorder and HC
Pae *et al*. 2004 [18]	South Korea	DSM-IV for SCZ	n = 47, M/F = 21/26, Mean age = 27.9 ± 6.8	Not specified	Antipsychotic-naive	Plasma	4.7 ± 1.3 mg/dL	5.3 ± 1.1 mg/dL	No differences in UA levels between first-episode SCZ patients and risperidone-treated chronic SCZ patients
Yao *et al*. 2010 [17]	USA	DSM-IV for SCZ or SZA	n = 25, M/F = 19/6, Mean age M/F = 21.4 ± 5.5/26.3 ± 10.6	M/F = 1.23 ± 1.29 years/2.43 ± 2.22 years	Antipsychotic-naive	Plasma	3.7 mg/dL	5.06 mg/dL	Possibly lower levels of UA in first-episode SCZ or SZA than in HC. No significant differences before vs after 4 weeks of antipsychotic treatment
Sarandol *et al*. 2015 [1]	Türkiye	DSM-IV for SCZ or BD	n = 26, M/F = 10/16, Mean age = 25.6 ± 7.0	Not specified	Antipsychotic-naive	Serum	5.41 ± 1.8 mg/dL	4.62 ± 1.46 mg/dL	No significant differences in UA levels between FEP patients and HC. No significant difference before vs after 6 weeks of treatment
Tao *et al*. 2020 [19]	China	DSM-IV for SCZ	n = 90, M/F = 44/46, Mean age = 21.5 ± 7.7	0.49 ± 0.53 years	Antipsychotic-naive	Serum	4.91 ± 1.47 mg/dL	4.6 ± 1.01 mg/dL	No significant differences in UA levels between first-episode SCZ patients and HC
Borovcanin *et al*. 2022 [9]	Serbia	ICD-10 for SCZ	n = 86, M/F = 36/50, Mean age = 33.64 ± 8.84	0.28 ± 1.93 years	Antipsychotic-naive	Serum	5.7 ± 2.96 mg/dL	5.59 ± 1.41 mg/dL	No significant differences in UA levels between FEP patients, SCZ in relapse and HC, neither before nor after 4 weeks of antipsychotic treatment
Jia *et al*. 2023 [20]	China	DSM-IV for SCZ	n = 67, M/F = 33/34, Mean age = 23.55 ± 6.07	1.30 ± 1.73 years	Antipsychotic-naive	Serum	4.91 ± 1.53 mg/dL	4.51 ± 1.18 mg/dL	No significant difference in UA levels between first-episode SCZ patients and HC
Wang *et al*. 2023 [21]	China	DSM-5 for SCZ	n = 148, M/F = 59/89, Mean age = 24.2 ± 6.62	Not specified	Antipsychotic-naive	Serum	4.57 ± 1.25 mg/dL	4.62 ± 1.18 mg/dL	No significant difference in UA levels between first-episode SCZ patients and HC. UA levels were significantly higher in first-episode SCZ patients after 24 weeks of antipsychotic treatment

BD, Bipolar disorder; DSM, Diagnostic and Statistical Manual of Mental Disorders; HC, Healthy controls; UA, uric acid; ICD, International Classification of Diseases; M/F, male/female; SCZ, Schizophrenia; SZA, Schizoaffective disorder; USA, United States of America.

In the study by Reddy *et al*. [14], blood samples were obtained from 40 HC and 43 neuroleptic- naïve FEP patients—31 non-affective FEP (SCZ or SZA) and 12 affective FEP (BD or depressive episode). Several individual antioxidants—UA, albumin and bilirubin—were compared between groups. Non-affective FEP patients had significantly lower UA, albumin and bilirubin as compared to HC. However, individual antioxidants were not significantly different between affective FEP patients and HC.

In Pae *et al*.’s study [18], the same individual antioxidants were analyzed—UA, albumin and bilirubin. The authors hypothesized that there might be a difference in the antioxidant system according to the ethnic background; they also aimed to examine the difference between drug-naïve first-episode SCZ and risperidone-treated chronic SCZ. This study showed a significant reduction in albumin and bilirubin levels in the patient group (n = 102) compared to HC (n = 68). Nevertheless, there were no differences in UA levels between first-episode SCZ patients (n = 47) and risperidone-treated chronic SCZ patients (n = 55).

Yao *et al*.’s study [17] approached the purine catabolism in non-affective FEP (SCZ or SZA), comparing levels of 6 purine-degraded products simultaneously in the plasma of a FEP group (n = 25) against an HC group (n = 30). Furthermore, the study analysed antipsychotic-treatment effect in the UA levels on the patient group. The antipsychotic treatment duration is similar in Yao *et al*.’s [17] and Pae *et al*.’s [18] studies—4 weeks. Values of both UA and guanine were lower in patient group than in HC. No significant UA differences were found before vs after antipsychotic treatment.

Sarandol *et al*.’s [1] study included FEP patients (n = 26) and HC (n = 25); like in Reddy *et al*.’s study [14], the neuroleptic-naïve FEP group included non-affective and affective patients. The evaluation of the antioxidative defense was extensive and included UA, albumin and bilirubin levels measurement—parameters also evaluated in the studies of Reddy *et al*. [14], and Pae’s *et al*. [18]. There was no significant difference in UA levels between FEP patients and HC group; there was also no significant difference in UA levels in FEP patients before vs after 6 weeks of treatment.

In Tao *et al*.’s study [19], samples from 90 first-episode SCZ patients and 70 HC were collected and analyzed to examine whether insulin resistance and oxidative stress are associated with cognitive impairment in drug-free first-episode SCZ, but no significant differences were found when compared to HC. In this context, UA levels were measured. Moreover, there was no correlation found between those biomarkers and the scores of Positive and Negative Syndrome Scale (PANSS).

Borovcanin *et al*.’s [9] study included 86 drug-naïve FEP patients, 45 patients with SCZ in relapse and 35 HC. Their UA levels were compared, before and after 4 weeks of antipsychotic treatment—the same timeframe considered in some of the studies above. There were no significant differences in UA levels between FEP patients, SCZ in relapse and HC, neither before nor after applied therapy.

Jia *et al*. [20] explored influencing factors of cognitive impairments and their relationships in drug naïve FEP. Sixty-seven patients with FEP were compared to HC and no significant difference in serum levels of UA were found between the two groups.

Wang *et al*. [21] studied if there were alterations in oxidative-stress related indicators in a group of 148 drug-naïve first-episode SCZ patients and 97 HC. This study also approaches the possible effect of antipsychotic medication, comparing peripheral biochemical indicators at baseline and after 24 weeks of treatment. No significant difference in UA levels between first-episode SCZ group and HC were found; however, UA levels were significantly higher in first-episode SCZ patients after antipsychotic treatment.

## 4. Discussion

This was the first systematic review specifically addressing UA levels in FEP, after a previous review about UA levels in SCZ patients had hypothesized that early phases of SCZ could be associated with lower levels of UA [10].

The eight studies included in this review were dissonant in finding a correlation between UA levels in FEP patients. Although in some studies FEP patients had lower UA levels in comparison to HC subjects [14, 17], in the overall such a tendency was not well demonstrated. It is however relevant to note that both studies describing reduced UA levels were based in North American samples, while Asian and European groups had unremarkable differences. Our systematic review included not only those 2 articles, but 6 other studies, 4 of which were published after 2020, with expanded data that is not supportive of the preliminary findings by He and colleagues [10]. Although our timeframe allowed for the inclusion of studies with less than 5 years duration of illness—the critical period traditionally defined by early psychosis, included studies involved patients in their FEP or within the first 2 years of SCZ.

The correlation between UA levels and psychotic disorders is important not only for its clinical implications but also in improving the understanding of psychotic disorders’ pathophysiology, supporting the hypothesis of an existing low antioxidant activity involved in such conditions, which could be translated with low UA levels, and decreased defense ability of brain cells in early psychosis [10]. A possible explanation for a pathological connection between oxidative stress and SCZ being present without correlation with UA levels might indicate that a non-enzymatic antioxidant system is still in place in drug-naïve FEP patients [21]. Other neurological conditions have also been associated with low UA levels and AODS impairment such as Parkinson’s disease [22], Alzheimer’s disease [23] and depression [24]. The evidence of a link between UA and cognitive function in other disorders has been explored in SCZ, namely in FEP. UA levels were found to be negatively connected with cognitive function in FEP patients, both before and after treatment, paving the way for behavioral and diet recommendations as relevant for influencing the cognitive dysfunction usually associated with SCZ [12].

The inconsistency of UA levels might also be due to other environmental factors such as genetic variants, renal function, gender, ethnicities, lifestyle or the diet of each individual [10]. The role of frequent unhealthy behaviors in individuals with psychosis such as poor diet, smoking, lack of physical exercise, and alcohol and drug abuse, could play a role in some findings involving UA levels [8]. Antipsychotic medication by itself could be a modifying factor, including its direct action on oxidative stress, namely reducing superoxide dismutase (SOD) activity, inflammation and even glucose metabolism; this underlines not only the complexity of antipsychotic’s therapeutic mechanisms, but also the importance of understanding the exact mechanisms of their influence on psychosis [21].

Although this systematic review provides some clinical evidence about UA levels in FEP individuals, several limitations should be held into account, besides the small number of available studies. First, there is a high heterogeneity across the studies that were reviewed due to factors such as duration of illness when UA levels were determined, measurement methods used or other factors such as ethnicity. This compromises both the possibility of finding significant results and the ability to generalize them. Second, several studies did not control to similar extent confounding factors, which may influence the association between SCZ and reduced UA. Third, both studies that found decreased UA levels had only 8% heterogeneity amongst the subgroup as these 2 studies come from the same research group, in the same USA location, are therefore cannot be considered fully independent studies. Additionally, we limited our search strategy to articles in English or Portuguese, languages for which the authors were fluent. All these limitations could compromise, for the time being, a solid assessment of the role of UA levels in early phases of psychosis.

## 5. Conclusions

There seems to be no consistent reduction of UA levels in patients with early phases of SCZ, namely FEP, and previously reported findings could be restricted to specific subgroups (e.g., American samples). Nonetheless, disruptions in UA metabolism among patients experiencing FEP represent an innovative and clinically significant avenue for research, possibly differentiating affective from non-affective FEP, being characteristic of the former, and therefore inconsistently found in cohorts where SCZ will be the predominant definitive diagnosis. Utilizing straightforward laboratory biomarkers in conjunction with clinical features could potentially minimize the time lapse between symptom onset and diagnosis. thereby impacting treatment decisions and prognostic assessments. To adequately investigate the relationship between UA levels (as an oxidative stress indicator) and FEP, particularly its potential as a diagnostic predictor, it is imperative to conduct longitudinal studies, preferably multicentric in nature, that prospectively track cohorts of individuals experiencing FEP.

## Data Availability

Data supporting this study is available from the corresponding author upon reasonable request.

## Supplementary Material

Supplementary material associated with this article can be found, in the online version, at https://doi.org/10.31083/AP44248.
